# Blunt Trauma and Diaphragm Injury in Children: An Analysis of the National Trauma Data Bank

**DOI:** 10.3390/children12020168

**Published:** 2025-01-29

**Authors:** Sammie Lai, Spencer Wilhelm, Robert Morden, Begum Akay, Nathan Novotny, Anthony Stallion, Pavan Brahmamdam

**Affiliations:** 1 Department of Pediatrics, University of Florida, Jacksonville, FL 32209, USA; 2 Department of Surgery, Corewell Health William Beaumont University Hospital, Royal Oak, MI 48073, USA; spencer.wilhelm@corewellhealth.org (S.W.); begum.akay@corewellhealth.org (B.A.);; 3 School of Medicine, Oakland University William Beaumont, Rochester, MI 48309, USA

**Keywords:** blunt trauma, diaphragm injuries, National Trauma Data Bank, pediatrics

## Abstract

**Background/Objectives**: Trauma is the leading cause of mortality and morbidity in children. Traumatic diaphragm rupture (TDR) is a rare but serious injury that can be difficult to identify. The current literature includes individual case reports and single-center case series only, which limits our ability to generalize those findings. The purpose of this study is to use the National Trauma Data Bank (NTDB) in order to examine the clinical outcomes of blunt TDR in the pediatric population. **Methods**: We included patients from 0 to 18 years of age with blunt TDR using the NTDB from 2007 to 2017. Patient characteristics and demographics, mechanisms of injury, concomitant diagnoses, procedures, and clinical outcomes were extracted from the NTDB. **Results**: In this study, we identified a total of 88 pediatric patients with blunt TDR. The most common mechanism of injury was motor vehicle accidents (65%). The majority of these blunt TDR injuries were observed in males (73%) with a mean age of 12. Fractures of the spine and ribs (49%) and lacerations of the lungs (42%) were some of the most prevailing associated injuries. Seventy percent of patients were admitted to the ICU. In-hospital mortality was 6%. **Conclusions**: Overall, TDR is relatively unusual among the pediatric population, but is associated with significant morbidity and mortality. Any significant trauma to neighboring organs—the spine, ribs, and lungs—should heighten awareness of potential diaphragm injury.

## 1. Introduction

Trauma is the leading cause of mortality and morbidity in children ages 1 through 18, and its associated injuries continue to be the prevailing reason for death in the pediatric population [1,2,3]. Annually, approximately 9.2 million injured children visit the emergency department in US hospitals [4]. Diaphragm injury is rare in both pediatric and adult populations, and it remains one of the most commonly missed diagnoses of trauma [5].

Traumatic diaphragm rupture (TDR) in the pediatric population is usually associated with other additional severe injuries [6]. Up to 8% of pediatric trauma patients who visited the emergency department were found to have concomitant diaphragm injuries [6,7,8,9]. The initial diagnostic approach to these injuries continues to be a challenge to pediatric surgeons because no single imaging modality is specific enough for the diagnosis [5]. It is important to diagnose and treat TDR early because it can lead to intestinal strangulation, sepsis, and death; and delayed diaphragm repair is associated with an increased rate of morbidity and mortality [5,6,7,8,9]. Initial diagnostic exams for diaphragm injury include chest radiography (CXR) and computerized tomography (CT) scans [10]. Radiologic findings of TDR include the presence of an elevated hemidiaphragm and the protrusion of intestines into the thoracic cavity [6,11,12]. Unfortunately, CXRs have revealed only 17% of all TDR cases in some studies [12,13,14,15]. A CT scan is more sensitive than a CXR in identifying diaphragmatic injury, but TDR in children can still be difficult to identify due to children’s flexible rib cages and their natural response to stress by swallowing air, which can blur images [6].

Both individual case reports and single-center case series have been published to summarize the frequency and mechanisms of injury, as well as the diagnosis and clinical outcomes of TDR [1,8]. However, wide variability exists within the literature, and the epidemiology in the pediatric population within the United States is not well characterized. The reported incidence varies between centers: from 0.8% to 8% within the pediatric trauma population [1,7,8,9]. Some centers reported TDR diagnosis via an initial chest radiography (CXR) to be as low as 17%, while others reported a rate of 85% [6,15,16]. Hence, a larger set of data is necessary to establish more comprehensive and conclusive statistics regarding TDR. In this study, our aim was to use the National Trauma Data Bank (NTDB) to query the instances of TDR in pediatrics within the US from 2007 to 2017. Specifically, we intended to learn more about the frequency, diagnosis, treatments, and clinical outcomes of TDR in children.

## 2. Materials and Methods

The NTDB was used in this retrospective study to compile data regarding pediatric TDR. It contains over six million trauma cases from 900 US hospitals [17]. Datasets from 2007 to 2017 were obtained from the Committee on Trauma of the American College of Surgeons. The inclusion criteria were patients aged between 0 and 18 with diagnoses of TDR from blunt injuries only. Patients with diaphragm injuries were identified using the Abbreviated Injury Scale (AIS) and the International Classification of Disease Ninth revision (ICD-9), diagnostic code 862.1, followed by age stratification. ICD-9 diagnostic codes 800–899 and procedure codes 0–99 were used to identify the associated injuries and operations, respectively. The appropriate National Trauma Data Set (NTDS) complication variables were also used to determine complications such as pneumonia, acute respiratory distress, and deep-space infection. The mechanism of injury was identified using the External Cause of Injury codes (E-code) 810–888. Other collected variables included demographics (age, gender, and race), Injury Severity Score (ISS), percent admitted to Intensive Care Unit (ICU), hospital length of stay, ICU length of stay, ventilator days, and mortality. Descriptive analysis was performed using a *t*-test, linear progression, and cross-tabulation. The primary outcome was to characterize the morbidity and mortality of pediatric patients with TDR in the NTDB. Secondary outcomes include mechanisms of injury, diagnostic approaches, length of stay, and complications.

## 3. Results

A total of 2114 pediatric patients were identified with TDR. Among them, 88 had blunt TDR. The mean age was 12 years, and 73% were male. The predominant race was Caucasian (64%), followed by Black or African American (19%) and other races (11%) that were not Asian or Native American. The most common blunt mechanisms were motor vehicle collisions (65%) and falls (25%) (Table 1).

An array of diagnostic procedures was performed upon admission of these patients. CT scans of the thorax and the abdomen (57%) were diagnostic tools frequently used to evaluate patients’ severity of injuries and need for surgeries. CT scans of the head and neck (35%) and X-rays of both the upper and lower extremities (16%) were also commonly used to assess for brain hemorrhage and bone fractures, respectively (Table 2).

Multiple thoracoabdominal injuries were found in pediatric patients with blunt TDR. The most prevailing associated injury was fracture of the spine and rib (49%), followed by thoracic injuries. The lungs and their neighboring anatomical structures were most frequently affected: lung injury (42%), pneumohemothorax (28%), hemothorax (18%), pneumothorax (8%), esophagus (3%), and heart (3%). Abdominal injuries were ranked third, with solid organs being most likely affected: liver (38%), spleen (33%), kidney (22%), large bowel (20%), small bowel (14%), stomach (6%), pancreas (5%), and adrenal glands (5%) (Table 3).

The majority of these patients underwent a procedural or surgical intervention. Intercostal catheters were placed in 65% of pediatric patients for pleural drainage. In addition, exploratory laparotomy and thoracotomy were used as both diagnostic procedures and treatments to evaluate and repair various organs’ damages. The prevailing operations were performed on the diaphragm, including suture of laceration (42%), non-specified repair (18%), and hernia repair (8%). Gastrointestinal operations were also performed for the small intestine (31%), large intestine (24%), and stomach (15%). Surgeries executed on the spleen and liver amounted to 16% and 9%, respectively (Table 4).

Overall, pediatric patients with blunt TDR had severe injuries. The ISS was 29.89 out of 75. Over half of the patients (70%) were admitted to the ICU with an average of 9 days spent in the unit. The average length of hospital stay was 12 days with an average of 9 ventilator days. The mortality rate was 6% (Table 5).

During these patients’ hospital stays, multiple complications were observed in 41 (47%) patients. The most frequent complications occurred in the respiratory system, including pneumonia (16%) and Acute Respiratory Distress Syndrome (10%). Infection was also identified, such as organ/space surgical site infection (10%), systemic sepsis (6%), and superficial surgical site infection (4%). Other complications noted include cardiac arrest (8%), unplanned return to the operating room (8%), and bleeding (6%). No catheter-associated infection, compartment syndrome, myocardial infarction, or pulmonary embolism were observed (Table 6).

## 4. Discussion

While outcomes from diaphragm injuries are well described in adults, they are not well characterized in the pediatric population [16]. Traumatic diaphragm rupture (TDR) in the pediatric population is relatively rare. In one single-center series, TDR was seen in only 8% of total pediatric trauma cases, with an increase in both morbidity and mortality due to a delayed diagnosis [6]. A recent review of the existing pediatric diaphragm trauma literature shows wide variation in epidemiology and clinical outcomes [5,6,9,17,18]. In addition, the majority of individual case reports and single-center case series describe incidents outside of the United States of America. A larger set of data is necessary to establish more comprehensive and conclusive statistics regarding TDR.

Our study is the first to present national statistics regarding the incidence, patterns, mechanisms, and outcomes of diaphragm injuries in children who were admitted to participating institutions in the NTDB from 2007 to 2017. Although the NTDB implements measures to safeguard data quality, it has been noted that the dataset is based on voluntary participation, and data may be missing [17]. Specific granular analysis regarding diagnostics, timing of imaging, complications, and cause of death are unavailable. Additionally, patients who passed away before arriving at the hospital are not included in the NTDB and those who are managed in outpatient settings are not included in the statistics [2]. Despite these constraints, this study provides valuable national-level insights into pediatric TDR.

Nonetheless, our findings on blunt TDR in the pediatric population are comparable to those in the adult population demonstrated by various studies, with some explainable differences. In both pediatric and adult populations, the most common mechanism of injury is motor vehicle collisions (65% vs. 63%) and there is a prevalence in males (73% vs. 68%) [16]. This is also noted in a recent systematic review of pediatric TDR, which noted an MVC as the most common mechanism of injury (46.5%) [18]. The most frequently associated injury with blunt TDR is fracture of the spine and/or ribs, although the adult population has a much higher rate (74%) of fractures than the pediatric population (49%) [19]. This difference is likely due to the flexibility of children’s ribs and spines. Pulmonary injuries are the second most common injuries in both children and adults. Liver (38%) and spleen (33%) injuries are third and fourth, respectively, in children, while this order is reversed for injuries to the spleen (45%) and liver (40%) in the adult population [16]. This difference likely occurs because children’s livers are located several centimeters below their rib cage, in contrast to adults, allowing the liver to be in direct contact with their abdominal wall. Without rib cage protection, the liver becomes an easy target during sudden acceleration and deceleration accidents. Studies also showed that posture, such as standing vs. sitting, can change the vulnerability of various abdominal organs [20]. These studies, along with our data on injury patterns, demonstrate the importance of the use of restraints and their proper fit to minimize organ damages during motor vehicle collisions.

Clinical management of blunt TDR in children is overall similar to that in the adult population, but our results show subtle differences in the diagnostic and surgical procedures between these two populations. Many studies on the adult population have shown that, traditionally, radiography is used as the initial screening test, followed by computerized tomography (CT) to minimize missed diagnoses [6,15,17,21]. In our study, however, radiography of the chest and abdomen was performed in only 14% of pediatric patients, while CT scans of the thorax and the abdomen were performed in 57%. We attributed this variability to the well-known low sensitivity of radiography in diaphragm injuries [6,15,17,21]. Another explanation is that these patients have a high ISS and are more likely to receive a CT scan of the chest and the abdomen due to the mechanism and associated injuries.

Once blunt TDR is identified, suture of laceration and/or repair are necessary. Laparotomy has been the gold standard for diagnosis and treatment in both pediatric and adult populations, and it was performed in 51% of the pediatric patients in our study. Also, thoracotomy (11%) and laparoscopy (9%) approaches have gained popularity in recent years [21]. Contemporary studies have shown that laparoscopy produces similar perioperative and postoperative outcomes in hemodynamically stable patients with blunt injuries [22]. On the other hand, one study suggests that thoracotomy is associated with prolonged operation times and an increase in mortality rate [19].

In the NTDB, we found that both pediatric and adult patients had severe injuries, with an ISS of 29 and 33, respectively [16]. However, the mortality rate was markedly lower in the pediatric population (6% vs. 20%), which may be related to decreased comorbidities in the pediatric population and is consistent with the most recent systematic review (11.3%) [16,18]. Our finding was consistent with multiple past studies, and they attributed the difference in mortality rate to aging [23,24,25]. These studies also showed that an increase in hospital length of stay and consultations was associated with an increase in mortality in the adult population [26]. Overall, the complications seen after TDR are similar in children and adults. The most prevailing complications include pneumonia and Acute Respiratory Distress Syndrome, which is likely related to the significant thoracic trauma needed to cause a diaphragm repair, as well as the need for intubation and ventilatory support in this population.

In contrast, several previous single-center studies have reported mortality rates as great as 30% to 40% from pediatric blunt TDR, which is significantly higher than the rate in our study (6%) [6,17,27,28]. Earlier studies looked at approximately 40–50 cases in a span of 10 years, but they were completed over 20 years ago. Barsness et al. described six cases between 1992 and 2002, and no deaths were recorded [17]. A significant difference in mortality between all studies, including the most recent ones, may be due to relatively current legislative changes such as the Child Passenger Safety Law (2006), as well as contemporary technological advances and an accumulative understanding of pediatric blunt TDR injuries [29].

According to the National Highway Traffic Safety Administration, the mortality rate of children in motor vehicle collisions has decreased by 27% since the passage of the Child Passenger Safety Law, which requires children to be placed in appropriate child restraints according to age and size [30]. These data are consistent with our results revealing that the overall mortality rate of pediatric TDR has decreased in the past decade by approximately 30%. However, the mortality rate from motor vehicle collisions has recently increased by 8%, from 1144 in 2015 to 1233 in 2016 [30]. In all fatal car accidents, 38% of children are unrestrained [30]. These data, along with our results showing the associated severe injuries, strongly affirm the importance of proper restraint use.

Trauma is the leading cause of mortality and morbidity in the pediatric population. Although TDR is rare, it is a serious injury associated with increasing mortality and morbidity. Since no single diagnostic approach has been established to detect diaphragm injuries, trauma providers should keep TDR in mind when there is significant thoracoabdominal blunt trauma and injuries to the thoracic cage, liver, or spleen. Any of these injuries should prompt trauma providers to consider potential diaphragm rupture. Early diagnosis of TDR may mitigate potential adverse outcomes. While the majority of the pediatric patients with TDR were restrained in motor vehicle collisions, it is still crucial to design and use optimal restraints for children.

## Figures and Tables

**Table 1:** Characteristics of patients with blunt TDR.

Characteristic	
Number of Patients	88
Age (y), Mean	12.43
Gender (%)	
Male	72.73
Female	26.14
Race of Patient (%)	
Caucasian	63.64
Black or African American	19.32
Asian	0
Native American	0
Other Race	11.36
Unknown/Not Recorded	5.68
Mechanism (%)	
Motor Vehicle Collisions	64.8
Fall	25.0
Struck by Object	5.7
Machinery Accident	3.4
Pedal Cycle Accident	1.1

The majority of blunt diaphragm ruptures were observed in 12-year-old Caucasian males, with motor vehicle collisions being the most common mechanism of injury.

**Table 2:** Diagnostic procedures used on patients with blunt TDR.

	Percent	Frequency
Abdomen		
Laparotomy	51.14	45
Computerized Tomography (CT)	30.68	27
Ultrasound	11.36	10
Laparoscopy	9.09	8
X-Ray	2.27	2
Thorax		
Computerized Tomography (CT)	26.14	23
X-Ray	11.36	10
Thoracotomy	11.36	10
Head, Neck, Face		
Computerized Tomography (CT)	35.23	31
Magnetic Resonance Imaging (MRI)	3.41	3
X-Ray	1.14	1
Electroencephalogram	1.14	1
Extremities		
X-Ray	15.91	14
Magnetic Resonance Imaging (MRI)	2.27	2
Heart		
Ultrasound	5.68	5
Magnetic Resonance Imaging (MRI)	1.14	1
Urinary System		
Computerized Tomography (CT)	1.14	1
Ultrasound	1.14	1
Pyelogram	1.14	1
Cystogram	1.14	1
Spine		
Magnetic Resonance Imaging (MRI)	3.41	3
X-Ray	1.14	1
Non-Specified		
Computerized Tomography (CT)	35.23	31
Ultrasound	5.68	5
Magnetic Resonance Imaging (MRI)	1.14	1

CT scans were most commonly performed on the head and neck, followed by the abdomen and thorax, to evaluate the severity of injuries. Laparotomy, laparoscopy, and thoracotomy are used as diagnostic procedures as well as part of the treatment for damaged organs. Imaging of the upper and lower extremities, heart, urinary system, and spine was rare compared to that of the head, neck, thorax, and abdomen.

**Table 3:** Associated injuries of patients with blunt TDR.

Associated Injuries	Percent	Frequency
Axial Skeleton and Central Nervous System Injuries		
Fracture of Spine and Rib	48.86	43
Intracranial Injury, Excluding Those with Skull Fracture	22.73	20
Fracture of Skull	17.05	15
Injury to Nerves and Spinal Cord	5.68	5
Thoracic Injuries		
Lung Injury	42.05	37
Pneumohemothorax	28.41	25
Hemothorax	18.18	16
Pneumothorax	7.95	7
Esophagus Injury	3.41	3
Heart Injury	3.41	3
Other Non-Specific Thoracic Injury	2.27	2
Bronchus Injury	0	0
Abdominal Injuries		
Liver Injury	37.5	33
Spleen Injury	32.95	29
Kidney Injury	21.59	19
Large Bowel Injury	20.45	18
Other Non-Specific Abdominal Injury	17.05	15
Small Bowel Injury	13.64	12
Stomach Injury	5.68	5
Pancreas Injury	4.55	4
Adrenal Gland Injury	3.41	3
Biliary Tract Injury	0	0
Pelvis Injuries		
Bladder Injury	2.27	2
Other Pelvic Organ Injury	1.14	1
Ureter Injury	0	0
Uterus Injury	0	0
Other Injuries		
Fracture of Extremities	28.41	25
Certain Traumatic Complications and Unspecified Injuries	10.23	9
Injury to Blood Vessels	7.95	7
Dislocation	2.27	2
Sprains and Strains of Joints and Adjacent Muscles	0	0

The most commonly associated injury was fracture of the spine and ribs, followed by injuries to the lungs, liver, and spleen. This pattern of associated injuries was consistent with data revealing motor vehicle collisions as the most common mechanism of injury.

**Table 4:** Surgical procedures on patients with blunt TDR.

	Percent	Frequency
Pleura		
Intercostal Catheter for Drainage	64.77	57
Pleurectomy	2.27	2
Suture of Laceration	2.27	2
Non-Specified Repair	2.27	2
Diaphragm		
Suture of Laceration	42.05	37
Non-Specified Repair	18.18	16
Repair of Hernia	7.95	7
Small Intestine		
Partial Resection	15.91	14
Anastomosis	5.68	5
Ileostomy and Enterostomy	5.68	5
Suture of Laceration	3.41	3
Large Intestine		
Suture of Laceration	11.36	10
Hemicolectomy and Transverse Colon Resection	10.23	9
Anastomosis	1.14	1
Colostomy	1.14	1
Spleen		
Total Splenectomy	14.77	13
Non-Specified Repair	1.14	1
Stomach		
Gastrostomy	7.95	7
Suture of Laceration	3.41	3
Gastroenterostomy Without Gastrectomy	2.27	2
Partial Gastrectomy	1.14	1
Liver		
Suture of Laceration	6.82	6
Non-Specified Repair	1.14	1
Partial Hepatectomy	1.14	1
Trachea		
Tracheostomy	9.09	8
Brain and Skull		
Ventriculostomy	4.55	4
Cranioplasty	1.14	1
Kidneys		
Complete Nephrectomy	3.41	3
Nephrostomy	1.14	1
Others		
Spinal Cord	3.41	3
Heart and Pericardium	3.41	3
Rectum	3.41	3
Lungs	2.27	2
Esophagus	2.27	2
Urinary Bladder	2.27	2
Pancreas	1.14	1
Appendix	1.14	1
Anus	1.14	1

The most frequent procedure was intercostal catheterization for pleural drainage. The most common procedure performed was the repair of diaphragms, followed by gastrointestinal procedures. Surgeries on solid organs, such as livers and spleens, were also observed.

**Table 5:** Injury severity and hospital outcomes of patients with blunt TDR.

Variable	
Number of Patients	88
Injury Severity Score (ISS)	29.89
Oxygen Saturation (%)	92.10
Admitted to the Intensive Care Unit (%)	70.45
Intensive Care Unit Length of Stay (Days)	8.92
Ventilator Days (Days)	9.42
Hospital Length of Stay (Days)	12.4
Mortality (%)	5.68

The overall injuries in this population were severe, with the majority of patients ending up in the ICU for over one week. The overall course of hospital stay was almost 2 weeks.

**Table 6:** Complications of patients with blunt TDR.

Complication	Percent	Frequency
Pneumonia	16.33	8
Acute Respiratory Distress Syndrome (ARDS)	10.2	5
Organ/Space Surgical Site Infection	10.2	5
Cardiac Arrest with Cardiopulmonary Resuscitation (CPR)	8.16	4
Unplanned Return to the Operating Room (OR)	8.16	4
Bleeding	6.12	3
Systemic Sepsis	6.12	3
Acute Kidney Injury	4.08	2
Decubitus Ulcer	4.08	2
Deep Vein Thrombosis (DVT)	4.08	2
Superficial Surgical Site Infection	4.08	2
Unplanned Intubation	4.08	2
Unplanned Admission to the ICU	4.08	2
Coagulopathy	2.04	1
Coma	2.04	1
Stroke/CVA	2.04	1
Urinary Tract Infection	2.04	1
Severe Sepsis	2.04	1

The most common complication was observed in the lungs, followed by surgical site infection. Major complications including cardiac arrest, sepsis, and stroke were observed, but there was no incidence of compartment syndrome, myocardial infarction, and pulmonary embolism.

## Data Availability

The data presented in this study are available through the National Trauma Database and can be made available by the authors on request.

## References

[1] Imahara S.D., Hopper R.A., Wang J., Rivara F.P., Klein M.B. (2008). Patterns and Outcomes of Pediatric Facial Fractures in the United States: A Survey of the National Trauma Data Bank. J. Am. Coll. Surg..

[2] Maerz L.L., Davis K.A., Rosenbaum S.H. (2009). Trauma. Int. Anesthesiol. Clin..

[3] WISQARS Data Visualization. https://wisqars-viz.cdc.gov:8006/lcd/home.

[4] Borse N., Gilchrist J., Dellinger A.M., Rudd R.A., Ballesteros M.F., Sleet D.A. (2008). CDC Childhood Injury Report: Patterns of Unintentional Injuries Among 0–19 Year Olds in the United States, 2000–2006: (572462009-001).

[5] Marzona F., Parri N., Nocerino A., Giacalone M., Valentini E., Masi S., Bussolin L. (2016). Traumatic diaphragmatic rupture in pediatric age: Review of the literature. Eur. J. Trauma Emerg. Surg..

[6] Brandt M.L., Luks F.I., Spigland N.A., DiLorenzo M., Laberge J.-M., Ouimet A. (1992). Diaphragmatic Injury in Children. J. Trauma-Inj. Infect..

[7] Koplewitz B.Z., Ramos C., Manson D.E., Babyn P.S., Ein S.H. (2000). Traumatic diaphragmatic injuries in infants and children: Imaging findings. Pediatr. Radiol..

[8] Ramos C.T., Koplewitz B.Z., Babyn P.S., Manson D., Ein S.H. (2000). What have we learned about traumatic diaphragmatic hernias in children?. J. Pediatr. Surg..

[9] Sharma L.K., Kennedy R.F., Heneghan W.D. (1977). Rupture of the diaphragm resulting from blunt trauma in children. Can. J. Surg..

[10] Okur M.H., Uygun I., Arslan M.S., Aydogdu B., Turkoglu A., Goya C., Icen M., Cigdem M.K., Onen A., Otcu S. (2014). Traumatic diaphragmatic rupture in children. J. Pediatr. Surg..

[11] Beauchamp G., Khalfallah A., Girard R., Dube S., Laurendeau F., Legros G. (1984). Blunt diaphragmatic rupture. Am. J. Surg..

[12] Bocchini G., Guida F., Sica G., Codella U., Scaglione M. (2012). Diaphragmatic injuries after blunt trauma: Are they still a challenge?. Emerg. Radiol..

[13] Gelman R., Mirvis S.E., Gens D. (1991). Diaphragmatic rupture due to blunt trauma: Sensitivity of plain chest radiographs. Am. J. Roent..

[14] Mirvis S.E., Shanmuganagthan K. (2007). Imaging hemidiaphragmatic injury. Eur. Radiol..

[15] Shapiro M.J., Heiberg E., Durham R.M., Luchtefeld W., Mazuski J.E. (1996). The unreliability of CT scans and initial chest radiographs in evaluating blunt trauma induced diaphragmatic rupture. Clin. Radiol..

[16] Fair K.A., Gordon N.T., Barbosa R.R., Rowell S.E., Watters J.M., Schreiber M.A. (2015). Traumatic diaphragmatic injury in the American College of Surgeons National Trauma Data Bank: A new examination of a rare diagnosis. Am. J. Surg..

[17] Barsness K.A., Bensard D.D., Ciesla D., Partrick D.A., Hendrickson R., Karrer F.M. (2004). Blunt Diaphragmatic Rupture in Children. J. Trauma Acute Care Surg..

[18] Theodorou C.M., Jackson J.E., Beres A.L., Leshikar D.E. (2021). Blunt Traumatic Diaphragmatic Hernia in Children: A Systematic Review. J. Surg. Res..

[19] Lim K.H., Park J. (2018). Blunt traumatic diaphragmatic rupture. Medicine.

[20] Hayes A.R., Gayzik F.S., Moreno D.P., Martin R.S., Stitzel J.D. (2013). Abdominal Organ Location, Morphology, and Rib Coverage for the 5th, 50th, and 95th Percentile Males and Females in the Supine and Seated Posture using Multi-Modality Imaging. Ann. Adv. Automot. Med..

[21] Chughtai T., Ali S., Sharkey P., Lins M., Rizoli S. (2009). Update on managing diaphragmatic rupture in blunt trauma: A review of 208 consecutive cases. Can. J. Surg..

[22] Lin H.-F., Chen Y.-D., Chen S.-C. (2018). Value of diagnostic and therapeutic laparoscopy for patients with blunt abdominal trauma: A 10-year medical center experience. PLoS ONE.

[23] Weaver A.A., Schoell S.L., Talton J.W., Barnard R.T., Stitzel J.D., Zonfrillo M.R. (2018). Functional outcomes of thoracic injuries in pediatric and adult occupants. Traffic Inj. Prev..

[24] Doud A.N., Weaver A.A., Talton J.W., Barnard R.T., Schoell S.L., Petty J.K., Stitzel J.D. (2015). Mortality Risk in Pediatric Motor Vehicle Crash Occupants: Accounting for Developmental Stage and Challenging Abbreviated Injury Scale Metrics. Traffic Inj. Prev..

[25] Schoell S.L., Weaver A.A., Talton J.W., Barnard R.T., Baker G., Stitzel J.D., Zonfrillo M.R. (2018). Functional outcomes of motor vehicle crash thoracic injuries in pediatric and adult occupants. Traffic Inj. Prev..

[26] Dülger D., Albuz Ö. (2020). Risk indices that predict in-hospital mortality of elderly patients. Turk. J. Med. Sci..

[27] Sharma A.K., Kothari S.K., Gupta C., Menon P., Sharma A. (2002). Rupture of the right hemidiaphragm due to blunt trauma in children: A diagnostic dilemma. Pediatr. Surg. Int..

[28] Annual Call for Data: National Trauma Data Bank (NTDB). American College of Surgeons. https://www.facs.org/quality-programs/trauma/tqp/center-programs/ntdb.

[29] Eslami M.H., Saadeddin Z.M., Rybin D.V., Avgerinos E.D., Eslami P.W., Siracuse J.J., Farber A. (2019). Trends and Outcomes of Pediatric Vascular Injuries in the United States: An Analysis of the National Trauma Data Bank. Ann. Vasc. Surg..

[30] Traffic Safety Facts. https://crashstats.nhtsa.dot.gov/Api/Public/ViewPublication/812491.

